# Effects of Nanoplastics on Freshwater Biofilm Microbial Metabolic Functions as Determined by BIOLOG ECO Microplates

**DOI:** 10.3390/ijerph16234639

**Published:** 2019-11-21

**Authors:** Lingzhan Miao, Song Guo, Zhilin Liu, Songqi Liu, Guoxiang You, Hao Qu, Jun Hou

**Affiliations:** Key Laboratory of Integrated Regulation and Resources Development on Shallow Lakes of Ministry of Education, College of Environment, Hohai University, 1 Xikang Road, Nanjing 210098, China; lzmiao@hhu.edu.cn (L.M.); gshhu60122@163.com (S.G.); lzl1993@hhu.edu.cn (Z.L.); liusq@hhu.edu.cn (S.L.); hjyyouguoxiang@hhu.edu.cn (G.Y.); Dquhao@163.com (H.Q.)

**Keywords:** nanoplastics, biofilms, metabolic functions, BIOLOG ECO microplate, AWCD, carbon source utilization

## Abstract

Nanoplastic (NP) contamination is becoming a pervasive issue as NPs, originating from microplastic particles, pose potentially harmful environmental impacts on aquatic ecosystems. The environmental hazards of NPs on microorganisms have been well documented in recent studies; however, little is known about their ecotoxicity effects on freshwater biofilms, which serve as important primary producers and decomposers and are highly connected with other ecosystem components. We investigated the effects of NPs on the microbial metabolic functions of freshwater biofilms in terms of carbon source utilization ability. Biofilm samples were collected, cultivated in a hydrodynamic flume for six weeks, and then exposed in polystyrene (PS) beads (100 nm in size) with different NP concentrations (1, 5, and 10 mg/L). BIOLOG ECO microplates were used to quantify carbon source utilization characteristics. The data were analyzed using average well-color development (AWCD), functional diversity indices, and principle component analysis (PCA). Results showed that the total carbon metabolic functions (represented by AWCD) remained constant (*p* > 0.05) with elevated NP concentrations, but some specific carbon sources (e.g., esters) changed in their utilization ability (*p* < 0.05). The microbial functional diversity (Shannon–Wiener diversity index, Simpson diversity index, and Shannon evenness index) was significantly reduced under 10 mg/L NPs (*p* < 0.05), indicating an inhibiting effect of NPs on biofilm metabolic diversity. This study examined NP ecotoxicity effects on microbial metabolic activities at the community level, but further studies are required to fully understand the mechanisms driving this change.

## 1. Introduction

The quantity of manufactured plastics has dramatically increased globally since the 1950s, reaching 359 million tons in 2018 [1]. Although there has been an increasing trend of plastic waste recycling recently, the vast majority of plastics (79% in 2015) still end up in landfills or are discarded in the environment [2]. Consequently, such inadequate waste management has paved the way for the formation of microplastics (MPs) (plastics less than 5 mm in size) through mechanical fragmentation, biodegradation, or product use such as in cosmetics and cleansers [3,4,5,6]. Entering freshwater networks in great quantities (0.12–387 items/m^3^) [7], MPs may induce negative impacts on aquatic flora and fauna such as phytoplankton, invertebrates, mollusks, and fishes [5,8,9,10]. Aquatic organisms can either directly ingest free MP particles from the water column and sediment [11] or via consuming contaminated prey, enabling MPs to accumulate along the food web [12]. Moreover, MPs can transform into nanoplastics (NPs) (diameter < 1 μm) through further fragmentation processes including UV radiation, mechanical abrasion, and biodegradation [3]. The ecological hazards from NPs should be addressed accordingly, taking into account the effects of their size differences from MPs [13]. Further, there is a lack of methods for detecting and quantifying NP concentrations, making the situation more problematic and unmanageable and emphasizing the urgency of understanding the fate and potential impacts of NPs [14].

Recent studies have demonstrated that NPs can profoundly alter aquatic organisms’ metabolic functions in various ways. They are more likely to induce toxicological effects in cells than are MPs since their smaller size allows them to permeate biological barriers and lipid membranes, though the majority of plastics, such as polystyrene (PS), are typically considered to be non-toxic [4,13]. NPs can damage membrane structures, affecting molecule diffusion and even gene expression [4,15]. A study on phytoplankton response demonstrated that NPs (100 nm) can first impair but then facilitate the reproduction of algae (*Chlorella pyrenoidosa*), as PS concentrations vary from 10 to 100 mg/L [16]. Researchers have also shown that the growth rate and cellular chlorophyll-a content of *Scenedesmus obliquus* decreases after exposure to NPs [17]. PS NPs (80 mg/L) can also inhibit the growth and ammonia conversion efficiency (ecological function) of the marine bacterium *Halomonas alkaliphila*, which induces increased extracellular polymeric substance production, which acts as a potential bacterial protective mechanism [18]. While the available literature has shed some light on NP toxicity effects on a wide range of freshwater organisms, the emerging risks for biofilm communities have not yet been investigated.

Once released into a freshwater environment, NPs may float in the water for months and then accumulate in benthic habitats where they interact with the microbial community [4,19]. It is still unclear how NPs influence microbial ecosystem functions at the community level. Biofilms, which consist of complex aggregates of microorganisms, including bacteria, archaea, algae, fungi, protozoa, and metazoa, serve as the “microbial skin” by colonizing on benthic surfaces and play a vital role in the primary production and biogeochemistry cycle of freshwater ecosystems [20,21]. The activities of algae, bacteria, and fungi are involved in the carbon transformation and biodegradation process [22]. For example, natural biofilms are capable of assimilating dissolved organic carbon originating from aquatic angiosperms in the overlying water, which results in a successful microbial community structure [23]. A similar biodegradable pattern of biofilms was also observed for synthetic organics such as *p*-nitrophenol [24]. Hence, distinct carbon source utilization by benthic biofilms may be used to interpret their metabolic functional characteristics and community structure to a certain degree. Considering that cell functions can be impaired by NPs in terms of gene expression, membrane structure, and cell viability [15], NPs may also damage metabolic activities of microorganisms living in biofilms, and biofilm carbon utilization functions may be simultaneously altered.

The aim of this study is to quantify the response of freshwater biofilms and their metabolic functions when they are exposed to different concentrations of NPs. It has been demonstrated that high concentrations of PS NPs induced oxidative damage of bacteria and affect their ecological functions [18,25]. Therefore, we hypothesize that NPs may act as a toxicant to biofilms, and the metabolic functions of biofilms might be influenced by NPs [4]. To verify this hypothesis, we collected biofilm from Xuanwu lake (Nanjing, China) as the community source and transferred the samples to a laboratory where a biofilm culture experiment was conducted. PS NPs were introduced for the exposure experiments because PS is one of the most abundant types of plastics that has been detected in freshwater aquatic environments. The carbon metabolic functions of the biofilm were investigated using BIOLOG ECO microplates.

## 2. Materials and Methods 

### 2.1. Characteristics of Nanoplastics

PS particles were purchased from DaE Science and Technology Co. Ltd. (Tianjin, China). The PS beads were evenly suspended in deionized water (diameter 100 nm, density 25 mg/cm^−3^). Once received, the PS solution was directly stored in a refrigerator (4 °C) until the experiment was executed.

The particle size distribution and surface charge of PS NPs used in this study were measured in Milli-Q water and a filtered experimental solution (used for biofilm cultivation) with a Zetasizer Nano ZSP instrument (Malvern Instruments, Malvern, UK) [26]. The morphology of the PS NPs was imaged using a scanning electron microscope (SEM) (Hitachi S-4800 SEM, Japan).

### 2.2. Biofilm Cultivation

The community of biofilms was collected from benthic rocks from Xuanwu lake in downtown Nanjing, East China (118.7837° E, 32.0692° N, height 9 m). Meanwhile, 50 L of freshwater from Xuanwu lake was also obtained as a potential source of biofilms. The samples were transferred to a laboratory and mixed in a dynamic ecological water tank (made with polymethyl methacrylate; length 4 m, width 0.3 m, and depth 0.3 m) to simulate natural conditions [27]. The tank was situated in a greenhouse to secure a stabilized microclimate (20 ± 0.5 °C). Hundreds of cobblestones (diameter 3–4 cm) were placed in the incubator tank to serve as substrates for the microorganisms. Halogen lamps (90–110 μmol m^−2^ s^−1^, light: dark = 12:12 h) were provided as light sources. Moreover, WC medium was added every 5 d to secure a balanced nutrition level to support microbial growth. The culture experiment was conducted for six weeks—after which, mature and stabilized biofilms were obtained (See Figure S1 and Figure S2) on the cobblestones and used in the exposure experiments [28].

### 2.3. Ecotoxicity Experiment

After six weeks of cultivation, the cobbles and their attached mature biofilms were carefully collected and transferred into microcosms (cylindrical plexiglass), each filled with 2.5 L of water (approximately 10 cm in depth) in a tank. The microcosms were placed in an indoor laboratory where a stabilized environment was secured (20 ± 0.5 °C, light: dark = 12:12 h). Approximately 30 cobbles were randomly transferred to each microcosm. To investigate the potential concentration effects of NPs, the PS solution was injected into the microcosms at three environmentally relevant concentrations (1.0 mg/L, 5.0 mg/L and 10.0 mg/L). The PS dosages chosen are higher than environmental concentration (predicted to be 1 μg/L) in order to investigate the potential effects of NPs on ecosystems in the future as the amount of plastic debris entering waters is estimated to increase [29,30]. Both the control (no PS injection) and each treatment contained three replicates. A propeller with an average rotation speed of 150 r/min was hung over each microcosm to simulate natural flow conditions and facilitate PS–biofilm contact. Artificial light was also offered throughout the exposure period (5 d).

At the end of exposure, the biofilms were carefully peeled off from the cobblestones using a sterile brush and transferred to flasks where they were blended using sonication (40 K Hz, 100 W, 1 min) and centrifugated in a centrifugal tube before use in further experimentation [31,32].

### 2.4. Community-Level Physiological Profiling

BIOLOG ECO Microplates served as an efficient tool to detect microorganism carbon source utilization abilities, which revealed the characteristics of their metabolic functions since different species are able to utilize different carbon sources [33,34]. The microplates consisted of 96 wells divided into three groups, with each group containing a blank well and 31 different sole carbon source (of 6 biochemical categories) wells, providing a triplicated experiment on one plate [35]. Further, a redox dye indicator was contained in each well to reflect the nicotinamide adenine dinucleotide (NADH) production from cell respiration. The absorbance of each well directly indicated the degree of carbon source utilization ability and was measured by average well-color development (AWCD).

For each group, 5 g of each biofilm sample was suspended in 90 mL of a lead sulfide (PbS) solution and vortexed for 2 min. In total, 1 mL of the mixture was collected and diluted in the PbS solution until the optical density (OD) value of each sample reached 0.05 at 590 nm [36]. Then, 125 μL of diluent from each group was added to the corresponding ecoplate wells and incubated 25 °C in the dark. The absorbance was measured at a wavelength of 590 nm every 24 h using a multifunctional enzyme label tester for 6 d.

The average well-color development can be calculated as:

(1) $$\mathrm{AWCD}=\overset{n}{\underset{i=1}{\sum }}\left(\mathrm{Ci}-R\right)/n$$

In Equation (1), Ci is the OD value of each well at 590 nm, *R* is the OD value of the control well, and n represents the number of wells. In addition, Ci and *R* values less than 0.06 are considered to be zero.

The number of carbon sources and the degree to which a microbial community can utilize them on one plate represent the metabolic potential of a microbial community. The metabolic diversity can be expressed by diversity indices based on AWCD. The indices below are calculated in this experiment.

Shannon–Wiener diversity index:

(2) H′=−∑Pi lnPi,Pi=(Ci−R)/∑(Ci−R)

*P*i represents the relative ratio of the absorbance value in the ith (1–31) well.

Simpson diversity index (D):

(3) $$D=1-\sum {P_{i}}^{2}$$

The Shannon–Wiener and Shannon indices are both widely applied in ecological studies to interpret the combined effects of richness and evenness.

Shannon evenness index:

E = H′/lnS(4)

S represents the total number of utilized carbon sources (31 carbon sources).

McIntosh index:

D = (N − U)/(N − √N)(5)

N is the total number of individuals in the sample and U is given by the expression:

(6) $$U=\sqrt{\sum n_{i}^{2}}$$

n_i_ is the quantity in the ith unit (Ci − R in this case), and the summation is undertaken over all the units (Ci − R).

When analyzing the data, principal component analysis (PCA) was performed using R to identify carbon source utilization patterns (CSUPs). PCA is a statistical procedure that illustrates correlated data by reducing dimensions.

## 3. Results and Discussion

### 3.1. Characterization of PS Beads

As displayed in Table 1, the size distribution of PS particles differed significantly when suspended in Milli-Q water or the experimental solution. The hydrodynamic diameters of PS were higher in the experimental solution than that in the Milli-Q water (*p* < 0.05). Further, the absolute value of the zeta potential was lower in the experimental solution (*p* < 0.05), indicating that the electrostatic repulsion between PS NPs was inhibited [37]. Previously, Sun et al. observed similar results for PS NPs suspended in distilled water and sterile seawater [38].

### 3.2. AWCD of All Carbon Sources 

**Figure 1:**
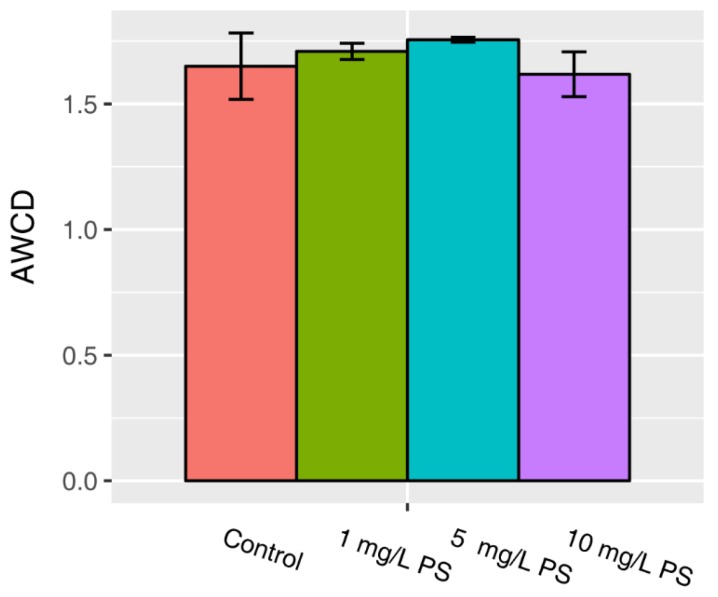
Average well-color development (AWCD) of all carbon sources. No significant difference (*p* > 0.05) was observed between the PS-introduced groups and the control.

The AWCD of all carbon sources reflects the capability of biofilms to assimilate all 31 types of carbon sources, revealing their level of metabolic activity. The AWCD of all carbon sources of the NP-treated biofilms are shown in Figure 1. No significant differences or clear trends were observed between the different concentrations of NP treatments. These results suggested that the total carbon utilization of freshwater biofilms was not significantly affected by increasing NP concentrations from 0 to 10 mg/L in this system.

### 3.3. AWCD by Biochemical Categories

**Figure 2:**
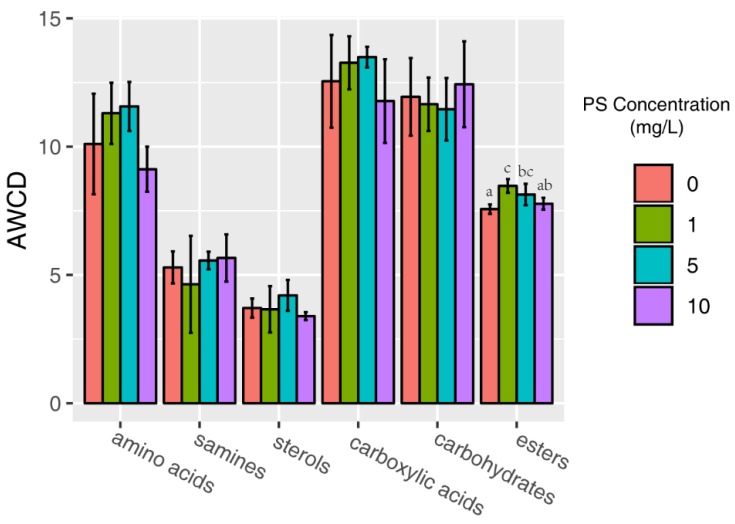
The AWCD by biochemical categories. Only the AWCD of esters reported significant differences (*p* < 0.05) between the experimental and control groups. The letters of each column represent a significant difference at *p* < 0.05.

All 31 carbon sources can be divided into six categories, including carbohydrates, amino acids, esters, sterols, amines, and carboxylic acids, based on biochemical properties and molecular composition, which paves the way to estimate metabolic functions in a physiologically relevant approach [39]. The extent of the utilization of specific carbon source categories by biofilms treated by NPs was analyzed and is displayed in Figure 2. In general, carbohydrates and carboxylic acids represented the two highest amounts of carbon sources utilized across all biofilm samples. The high consumption of carbohydrates has also been previously reported in the literature [39,40]. Sterols and amines were consumed the least of the six categories after NP exposure.

The carbon utilization characteristics (*p* > 0.05) of carbohydrates, amino acids, sterols, amines, and carboxylic acids were similar. The AWCD of esters, however, differed significantly from the other four test groups. The effect on ester microbial metabolism by NP beads displayed a concentration-dependent pattern. The usage of esters by biofilm samples exposed to 1 and 5 mg/L NP environments was significantly greater than that of esters from the control group (*p* < 0.05)—by 12% and 8%, respectively. Despite the fact that the metabolic activities of the esters in the 1 and 5 mg/L samples were higher than those in the NP-free environment, the level of activity decreases to the control level in the 10 mg/L ambient NPs group, illustrating that the utilization pattern of esters by biofilm communities might be attributed to hormesis effects with low dose stimulation and high dose inhibition. It can be inferred from the results that the ester-consuming microorganisms inhabiting biofilms responded more sensitively than other species, in terms of metabolic activity, to the elevation of NP concentration.

### 3.4. AWCD by Specific Carbon Source 

**Figure 3:**
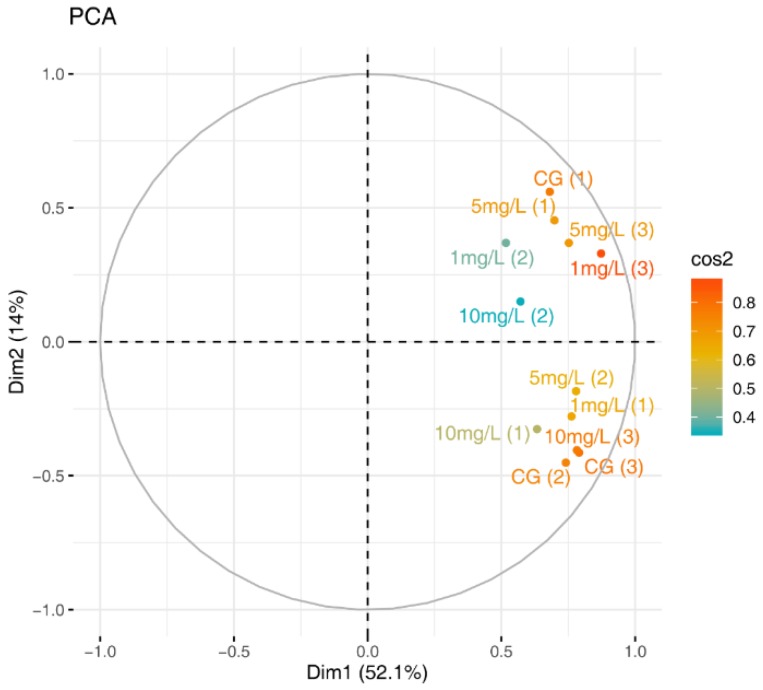
Nanoplastic (NP)-introduced aquatic PCA ordination showing the microbial community carbon source utilization patterns (CSUPs). CG represents the control group. The cos2 values are used to estimate the quality of the representation. The closer a variable is to the circle of correlations, the better its representation on the factor map and the more important it is for interpreting these components.

The AWCD by specific carbon source reflects the relative degree of usage of the 31 typical carbon sources by the freshwater periphyton exposed in the PS-introduced environment. The carbon source utilization patterns (CSUPs) of freshwater periphyton were compared using the PCA and the carbon sources. As shown in Figure 3, all four groups were situated on the right corner of Dimension 1. The PCA indicated that there was no significant difference between the CSUPs for periphyton after exposure to NPs in this study.

**Figure 4:**
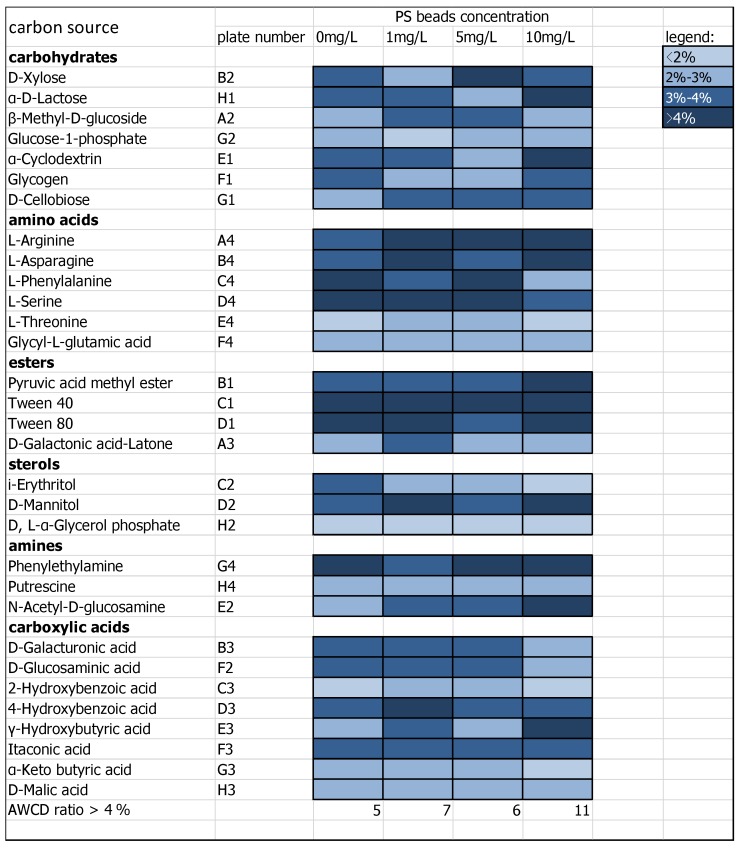
The carbon utilization pattern of 31 specific carbon sources by the freshwater biofilm microbial community exposed to an NP-introduced environment. Light blue indicates low carbon source usage by the microbial community and darker shades of blue indicate higher usage.

The changes in the carbon sources of the periphyton are displayed in Figure 4. The chart reports that the carbon source utilization ratio by biofilms varies dramatically both for different carbon sources and aquatic environments treated with different PS particle concentrations. The dividing carbon source utilization ratio in this investigation was 4% [41].

The results showed that the number of high-utilization carbon sources (AWCD ratio exceeds 4%) in PS-introduced groups was higher than that in the control (5). The 10 mg/L PS concentration treatment displayed the greatest number of high-utilization carbon sources (11), distinguishable from the low-level ambient PS (1 mg/L) condition (7) and the medium-level ambient PS (5 mg/L) condition (6) observations. As such, the “AWCD gap” between the high-utilization carbon sources and other carbon types within the high-ambient PS level (10 mg/L) condition was clearer than that of the other three groups, since the overall AWCD shows no significant difference among the four groups. For specific carbon sources, Tween 40 serves as the only carbon source where each relative AWCD exceeds 4%, indicating high metabolic activity by the microbial communities in all four conditions. D, L-α-Glycerol phosphate is the carbon source that was not utilized (relative AWCD < 1%) by freshwater biofilms, and the consuming level of Glycyl-l-glutamic acid remained unchanged in the three PS-contaminated environments compared to the control. All other carbon sources changed in utilization to various degrees. The following carbon sources increased dramatically in utilization level in the 10 mg/L PS concentration sample compared to the control *N*-Acetyl-d-glucosamine, α-d-Lactose, and γ-Hydroxybutyric acid. The following carbon source utilization levels decreased dramatically in the 10 mg/L condition compared to the control: i-Erythritol, l-Phenylalanine, and d-Glucosaminic acid.

The utilization of *N*-Acetyl-d-glucosamine by the microbial community increased considerably by 43% from 1.522 in the control to 2.179 in the 10 mg/L PS group. *N*-Acetyl-d-glucosamine acts as the monomer of fungal, bacterial and chitin cell walls and serves as a major component of invertebrate exoskeletons [42]. The surge of *N*-Acetyl-d-glucosamine consumption with increased NP concentrations indicates an outbreak of organisms that can incorporate *N*-Acetyl-d-glucosamine, which may be due to NPs causing the collapse of cell walls and invertebrate mortality. This environmental stress may not eliminate certain species but changes in material production and biodegradation patterns can be expected. Furthermore, the death of the invertebrates may trigger a trophic cascade due to their leading trophic levels in the freshwater biofilm ecosystem, and these effects may be combined with other environmental stresses such as NPs. Further, the significant increase in α-d-Lactose by 36% suggests that the microbial community has the ability to consume lactose with an increased PS concentration. One possible explanation for this is that the high NP concentration stimulated lactose production.

The sharp reduction in AWCD of i-Erythritol by 68% with the increase in PS concentration from 0 to 10 mg/L indicates that i-Erythritol-consuming species abundance declined considerably with an increase in NP concentration, which might stem from the direct inhibition effects of NPs on the activities of those species or be due to the growing i-Erythritol shortage in the ecosystem. As i-Erythritol is mainly distributed in plant species, and it can be inferred that the activities of phototrophs such as algae and photosynthetic bacterium were impaired by the NPs. Considering algae growth is highly dependent on light availability, such an NP–cell interaction likely predominately takes place on the photosynthetic layer (outer layer) of biofilm structures.

The concentration AWCD pattern can be elucidated in three possible ways. First, a high PS concentration habitat could favor a certain community of periphyton species whose preferred carbon sources are examined and shown here. These species are more physically tolerant of PS particle impairment than their counterparts, which results in the enhancement of their survival rate. Second, the vertical stratification nature of freshwater periphytons may enable inner layer microorganisms to be protected from high NP pressure environments where outer layer microorganisms such as phototrophs suffer. Therefore, the oxidative stress, membrane-penetration rate, chemical communication interference, and other adverse impacts induced by NPs could vary among the microbes inhabiting different biofilm layers [18,43]. Third, organisms jeopardized by high NP environments may die; the structures of the dead microbes can then serve as new carbon sources for adjacent microbes. In short, the cumulative effects induced from these three explanations creates a new niche in different NP concentrations, providing opportunities for propagules or spores in dormancy to germinate and, consequently, reshape the benthic community structure.

### 3.5. Microbial Metabolic Functional Diversity

Microbial metabolic functional diversity (alpha diversity) was calculated then presented in terms of the Shannon–Wiener diversity index (**H′**), Simpson diversity index (**D**), Shannon evenness index (**E**), and McIntosh index (***D***), reflecting the carbon source utilization heterogeneity of biofilms and elucidating the community structure to a certain degree. The Shannon–Wiener diversity and Simpson diversity indices are linearly related to the relative abundance of the different carbon sources (evenness) and the log_2_ of the number of utilized carbon sources (richness) while the Shannon evenness index was only influenced by evenness.

As shown in Table 2, the metabolic functional diversity of the biofilm community displayed distinct patterns for different PS bead concentrations and different alpha diversity indices. For the Shannon–Weiner and Simpson indices, there were no significant differences between biofilm samples treated with 1 and 5 mg/L PS concentrations, while the two indices were significantly lower for the 10 mg/L PS group than for the control group, suggesting a negative PS NP effect. As the metabolic richness diversity is similar among all groups (the number of carbon sources consumed is the same), the patterns found in the Shannon–Wiener and Simpson indices were also found in the Shannon evenness index. For example, only the 10 mg/L PS concentration group induced a significant decrease in evenness compared to the control group (*p* < 0.05), while the other groups’ evenness remained unchanged. The McIntosh diversity remained constant five days after the PS NP treatment regardless of the PS concentration. This result not only coincided with the AWCD specific carbon source results, but a previous study also confirmed that NP effects on community composition peaked when the highest NP concentration was introduced [17]. This may be because the highest PS–PS or PS–cell interaction opportunities could be reached when the PS dosage was the highest, indicating an amplification effect. The low diversity index of the 10 mg/L PS beads group suggested a phase shift in the microbial community structure, allowing a certain group of species to become more predominant while other species lost their niche.

## 4. Conclusions

In this study, we demonstrated the potential ecotoxicity effects of NPs in freshwater systems on the carbon metabolic activity of biofilm communities in a five-day period. The results were measured by BIOLOG ECO microplates to investigate the metabolic patterns, and we found that the general AWCD did not change in the NP-introduced environments. However, clear differences in metabolic characteristics were elucidated for six biochemical categories and 31 specific carbon sources, indicating that the consumption preference changed in the microbial community. The metabolic functional diversity indices results were in accordance with results from our high-utilization carbon source experiment and previous research, suggesting that the inhibition of alpha diversity or a metabolism shift mainly occurred in the highest NP concentration group which may be related to the amplification effect and increased effect on microbial activities. Further studies are required to understand how biofilm communities respond to aquatic NP particles in long-term exposure (more than 5 days) considering biofilms serve as important primary producers and decomposers in aquatic ecosystems.

## Figures and Tables

**Table 1:** Particle size distribution and zeta potential of polystyrene beads.

Primary Particle Size	Particle Size Distribution (nm)		Zeta Potential (mV)	
	Milli-Q Water	Experimental Solution ^a^	Milli-Q Water	Experimental Solution
100 nm	129 ± 34	569 ± 124 *	−39.4 ± 3.9	−19.4 ± 3.5 *

^a^ The experimental solution used here was filtered through a 0.22 μm membrane. * An asterisk indicates a significant difference in average diameter and zeta potential for the polystyrene (PS) beads in the experimental solution compared with those in Milli-Q water (*p* < 0.05).

**Table 2:** The metabolic functional diversity indices indicating that significant differences only occurred in the 10 mg/L NP group compared to the control in terms of H’, D, E and *D*. H’ stands for Shannon–Wiener diversity index, D stands for Simpson diversity index, E stands for Shannon evenness index, *D* stands for McIntosh index. Data in the table are the mean ± variance, *n* = 3. Using Duncan’s multiple range test of diversity indices separately, different letters (a/b) in each index represent the significant difference at *p* < 0.05.

PS Concentration	H′	D	E	D
0 mg/L	3.36 ± 0.012a	0.964 ± 0.001a	2.275 ± 0.008a	0.838 ± 0.028a
1 mg/L	3.347 ± 0.017a	0.963 ± 0.001a	2.281 ± 0.01a	0.82 ± 0.012a
5 mg/L	3.376 ± 0.009a	0.964 ± 0a	2.271 ± 0.01a	0.815 ± 0.003a
10 mg/L	3.318 ± 0.041b	0.961 ± 0.002b	2.239 ± 0.021b	0.832 ± 0.01a

## Supplementary Materials

The following are available online at https://www.mdpi.com/1660-4601/16/23/4639/s1, Figure S1: The photograph of biofilms cultured for six weeks in the hydrodynamic flume in this study, Figure S2: Dry weight of biofilms (g/m2) after colonization (Day 0). The dry weight became stable since Day 36. Mature and stabilized biofilms were obtained on Day 43 to conduct ecotoxicity experiment.
