# Supplementary material for: Effects of Nanoplastics on Freshwater Biofilm Microbial Metabolic Functions as Determined by BIOLOG ECO Microplates
Source: Int J Environ Res Public Health. 2019 Nov 21;16(23):4639. doi: 10.3390/ijerph16234639 (PMC6926673; doi:10.3390/ijerph16234639)
Supplement: Supplementary file 1 [file ijerph-16-04639-s001.pdf]

Supporting Information for

Lingzhan Miao, Song Guo, Zhilin Liu, Songqi Liu, Guoxiang You, Hao Qu and Jun Hou *

Key Laboratory of Integrated Regulation and Resources Development on Shallow Lakes of Ministry of Education, College of Environment, Hohai University, 1 Xikang Road, Nanjing 210098, China; lzmiao@hhu.edu.cn (L.M.); gshhu60122@163.com (S.G.); lzl1993@hhu.edu.cn (Z.L.); liusq@hhu.edu.cn (S.L.); (hjyyouguoxiang@hhu.edu.cn (G.Y.); tsuhao_hhu@qq.com (H.Q.)

* Correspondence: hhuhjyhj@126.com or hjy_hj@hhu.edu.cn; Tel.: +86-25-83787332

There are 2 pages in this SI, containing 2 figures.

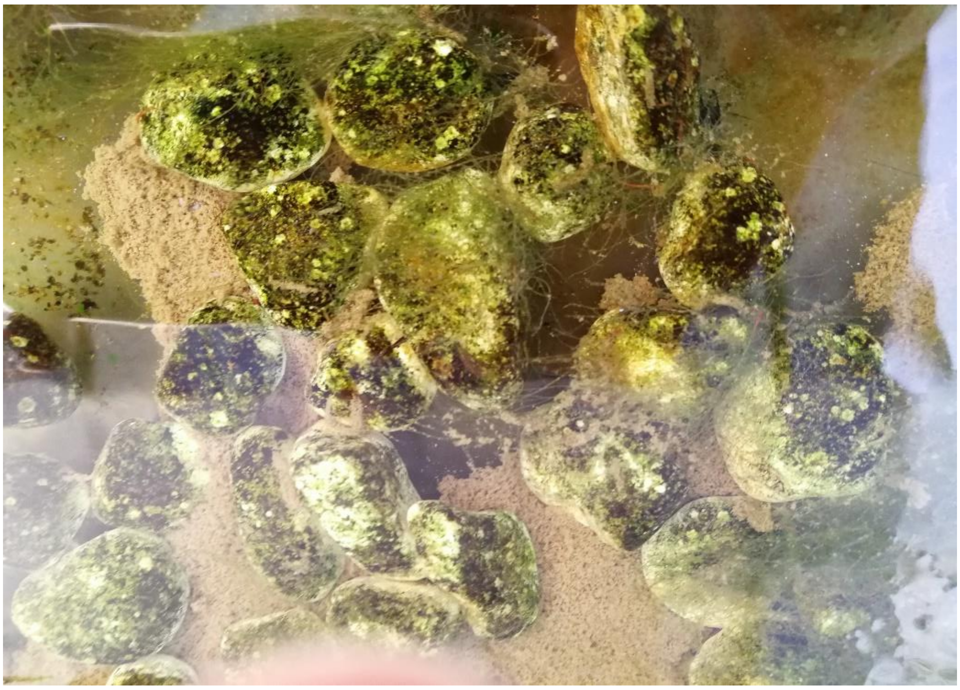

Figure S1. The photograph of biofilms cultured for six weeks in the hydrodynamic flume in this study.

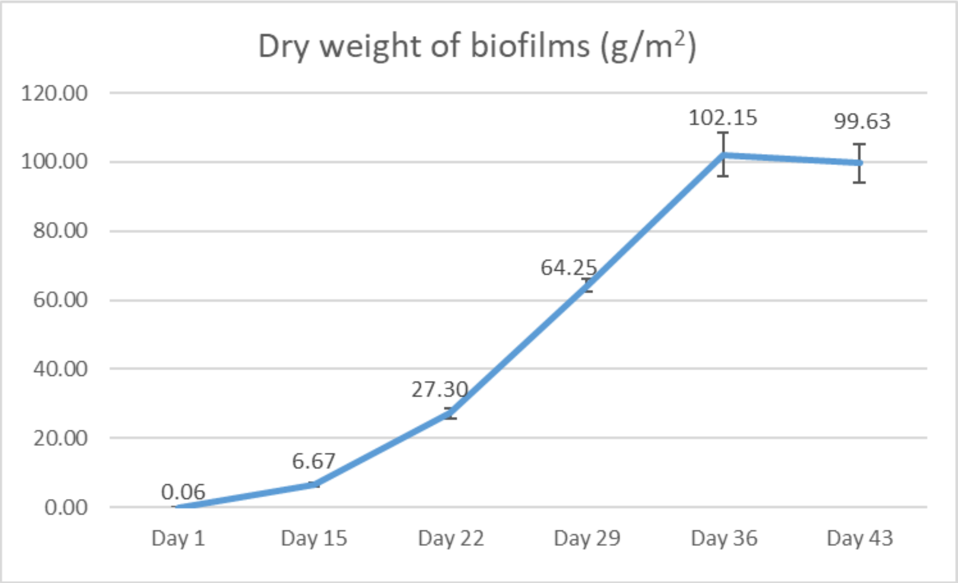

Figure S2. Dry weight of biofilms (g/m^2) after colonization (Day 0). The dry weight became stable since Day 36. Mature and stabilized biofilms were obtained on Day 43 to conduct ecotoxicity experiment.
